# Leveraging machine learning predictive biomarkers to augment the statistical power of clinical trials with baseline magnetic resonance imaging

**DOI:** 10.1093/braincomms/fcab264

**Published:** 2021-11-03

**Authors:** Carolyn Lou, Mohamad Habes, Nicholas A Illenberger, Ali Ezzati, Richard B Lipton, Pamela A Shaw, Alisa J Stephens-Shields, Hamed Akbari, Jimit Doshi, Christos Davatzikos, Russell T Shinohara

**Affiliations:** 1 Penn Statistics in Imaging and Visualization Center, Department of Biostatistics, Epidemiology, and Informatics, University of Pennsylvania, Philadelphia, Pennsylvania, 19104, USA; 2 Department of Biostatistics, Epidemiology, and Informatics, University of Pennsylvania, Philadelphia, Pennsylvania, 19104, USA; 3 Center for Biomedical Image Computing and Analytics, Department of Radiology, University of Pennsylvania, Philadelphia, Pennsylvania, 19104, USA; 4 Department of Neurology, Albert Einstein College of Medicine, New York City, New York, 10461, USA

**Keywords:** machine learning, clinical trials, neuroimaging

## Abstract

A key factor in designing randomized clinical trials is the sample size required to achieve a particular level of power to detect the benefit of a treatment. Sample size calculations depend upon the expected benefits of a treatment (effect size), the accuracy of measurement of the primary outcome, and the level of power specified by the investigators. In this study, we show that radiomic models, which leverage complex brain MRI patterns and machine learning, can be utilized in clinical trials with protocols that incorporate baseline MR imaging to significantly increase statistical power to detect treatment effects. Akin to the historical control paradigm, we propose to utilize a radiomic prediction model to generate a pseudo-control sample for each individual in the trial of interest. Because the variability of expected outcome across patients can mask our ability to detect treatment effects, we can increase the power to detect a treatment effect in a clinical trial by reducing that variability through using radiomic predictors as surrogates. We illustrate this method with simulations based on data from two cohorts in different neurologic diseases, Alzheimer’s disease and glioblastoma multiforme. We present sample size requirements across a range of effect sizes using conventional analysis and models that include a radiomic predictor. For our Alzheimer’s disease cohort, at an effect size of 0.35, total sample size requirements for 80% power declined from 246 to 212 for the endpoint cognitive decline. For our glioblastoma multiforme cohort, at an effect size of 1.65 with the endpoint survival time, total sample size requirements declined from 128 to 74. This methodology can decrease the required sample sizes by as much as 50%, depending on the strength of the radiomic predictor. The power of this method grows with increased accuracy of radiomic prediction, and furthermore, this method is most helpful when treatment effect sizes are small. Neuroimaging biomarkers are a powerful and increasingly common suite of tools that are, in many cases, highly predictive of disease outcomes. Here, we explore the possibility of using MRI-based radiomic biomarkers for the purpose of improving statistical power in clinical trials in the contexts of brain cancer and prodromal Alzheimer’s disease. These methods can be applied to a broad range of neurologic diseases using a broad range of predictors of outcome to make clinical trials more efficient.

## Teaser

Historical controls are a potentially rich source of data that can enhance the power of clinical trials if incorporated with appropriate methodological care. The results of Lou et al. show that high predictive accuracy from radiomic predictors can enhance this power in clinical trials that collect baseline imaging.

## Graphical abstract

**Graphical Abstract:**
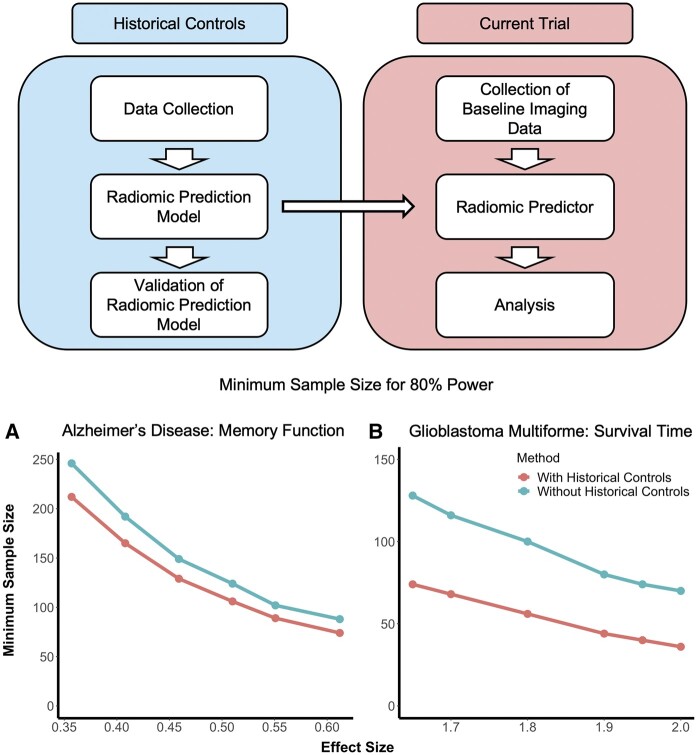

## Introduction

The power of a clinical trial is the probability of detecting a statistically significant difference between treatment groups under a set of assumptions. Power increases as the magnitude of the true difference in outcomes between treatment groups increases, as the accuracy of measurement for the outcome measure increases, and as sample size increases.^1^ When a treatment effect exists, failure to detect a statistically significant difference between treatment groups can occur as the result of a myriad of reasons, including small treatment effect, poor measurement of the primary outcome, inadequate sample size (underpowered studies) or treatment effect heterogeneity.^2–5^ Failure is more likely in studies of relatively rare neurologic diseases including glioblastoma multiforme (GBM) because enrolling large samples is difficult, but failure also occurs in more common diseases with substantial biological heterogeneity and unstable outcome measures, such as Alzheimer’s disease.^6–8^

Despite many long and expensive trials, no disease modifying drug for Alzheimer’s disease has been approved.^9^ Phase III trials for GBM have been more successful, but treatment efficacy has been modest, with an improvement in median survival of only 7 months (8‒15 months) for patients in the treatment arms in 44 different trials.^10^^,^^11^ Similar explanations have been proposed for the failure of trials of these two diseases, including biological heterogeneity, selection of ineffective treatments based on incomplete understanding of disease biology, starting treatment too late in disease development, incorrect drug doses and unreliability of the primary outcome measurements.^12^^,^^13^ All of these explanations may contribute to a reduction in the magnitude of the treatment effect. If the expected treatment benefit is overestimated, the study will be underpowered.

Traditionally, trials rely on empirical data from previously conducted studies (often phase II trials, if available) to estimate sample size requirements to achieve a particular level of power (i.e. 80%, 90%). These traditional methods for estimating sample size requirements rely on group mean data and calculating sample size requirements based on population average treatment effects. When historical control data are used, statisticians use methods such as pooling or Bayesian modelling, which also rely on group-level analyses.^14^^,^^15^ Newer high-dimensional predictors such as neuroimaging or genomic data offer the opportunity to include individualized predictions. This allows for a more precise evaluation of the treatment effect for each person through comparison of their observed outcome with their predicted outcome, rather than relying on a group-level effect that determines average outcome. Because of this more precise evaluation, the residual variance decreases and thus the power to detect this treatment effect increases.

In this study, we introduce the concept of using individualized evaluation of treatment effects with neuroimaging biomarkers and provide a framework for practically incorporating this approach into future clinical trials of neurologic disease when baseline imaging is available. We show that machine learning tools can provide individualized predictions for patients with Alzheimer’s disease and GBM, which in turn can be used to inform sample size calculations with individualized estimates of clinical outcome in a trial. This methodology can substantially improve statistical power for detecting treatment effects, or alternatively, reduce the sample size needed to achieve the same power in a clinical trial.

## Materials and methods

Our method relies on access to two sets of data: (i) a current clinical trial designed to study an outcome of interest and (ii) a previously observed cohort of similar subjects treated according to the current standard of care with data on the outcome of interest. We narrow our focus in this work to radiomic predictors and associated studies, so we assume that imaging data have been gathered at study enrolment for both sets of trials. In both of our disease applications, imaging data are regularly obtained through standard course of care, either for exclusion of other pathologies or for diagnosis itself. The techniques proposed here are also directly applicable to other -omic modelling scenarios, and generally, to any predictive marker of standard of care outcome.

**Figure 1:**
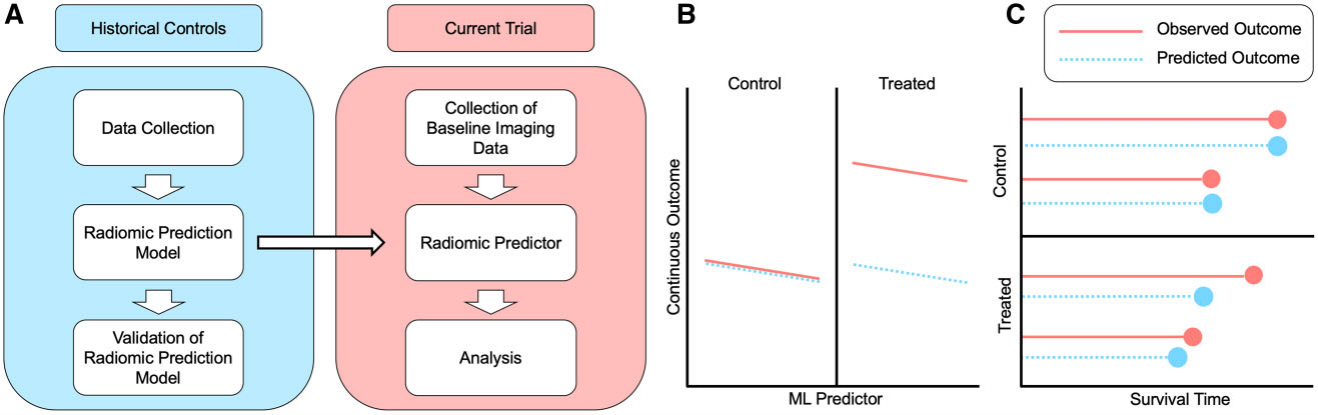
**Method visualization and description.** (**A**) Workflow for implementing the proposed method in a new clinical trial. **B** (continuous) and **C** (survival outcome): Schematic diagram for individualized predictions that are generated for each person in the current trial, where the solid red lines indicate observed outcome for the participants of the current trial and the dashed blue lines indicate predicted outcome for those participants had they not been treated. (**B**) illustrates the method for continuous outcomes, where the left side represents the outcomes of those randomized to the control arm and the right side represents the outcomes of treated participants. The predicted outcome values (dashed blue lines) for the control units had they not been treated would be exactly what they are observed to be (solid red lines), while the predicted outcome values (dashed blue lines) for the treated units had they not been treated are different from the observed outcome (solid red lines). (**C**) illustrates the analogous mechanism for survival outcomes, where the predicted survival times for the control units (dashed blue lines) are the same as the observed survival times (solid red lines), whereas the predicted survival for the treated individuals are lower than the observed survival times. Our method capitalizes on these differences to augment statistical power.

We aim to show that previously developed and validated radiomic prediction models, which summarize imaging patterns that predict future clinical outcomes of interest, can in some cases result in improved statistical power for detecting treatment effect (Fig. 1). These outcomes of interest can be endpoints such as response to treatment, patient survival, or progression-free survival. The model, which is built based on a historical cohort, can then be used in conjunction with data collected from the current trial to generate individualized values of the radiomic score for each of the current participants. These individualized scores represent predicted values for the outcomes of the treated individuals in the current trial had they instead been assigned to the control group. The incorporation of these predicted values as a covariate in the final analysis of the current trial lends power to the detection of the effect of a treatment by modelling the inter-subject variability in the outcome in terms of baseline heterogeneity represented in the baseline imaging.

In practice, this could be done by using a model developed for a previously validated radiomic predictor, applying it to data from a current trial of interest, and then incorporating the newly derived values of that radiomic predictor as a covariate in the study analysis. This would reduce uncertainty in the estimate of the overall treatment effect and therefore increase statistical power to detect a treatment effect.

To investigate the advantage of this approach, we implemented our models in two scenarios motivated by two different disease areas (GBM and Alzheimer’s disease). To better approximate real-life clinical trial performance, we use radiomic and outcome data from two observational studies to generate hypothetical study data, where the first focuses on the continuous outcomes of cognitive decline in prodromal Alzheimer’s disease and the second on the survival after diagnosis with GBM. With these studies, we performed plasmode simulations, where we randomly split our observational data into a theoretical historical cohort and a theoretical trial cohort. We then simulate effects in a randomly selected subset, corresponding to one arm, of the trial cohort. We then compare the statistical power of our proposed approach with the classical modelling approach that does not include radiomic prediction-based modelling.

We also performed simulations with fully synthetic data, where the populations were generated to be homogeneous except for random error and treatment status. Code for both the synthetic data simulations and plasmode simulations are available on our GitHub (https://github.com/carolynlou/hcct).

### Data

For our analyses, we relied only on observational data. These data were obtained from ADNI and the University of Pennsylvania for our Alzheimer’s disease and GBM studies, respectively.^9^^,^^16^ There was no missingness in either of the two datasets, and no patients dropped out before baseline imaging data could be collected. Our studies of performance in a clinical trial setting were based on plasmode simulations, where we artificially generated hypothetical trial data from our observational data.

In our first case study, we focussed on therapeutic trials for prevention of Alzheimer’s disease, in which the primary outcome is typically longitudinal cognitive change. Here, we simplified this outcome and quantified cognitive change as the difference between memory score measured 2 years from baseline and memory score measured at baseline. We used a predictive model, called the SPARE-AD score, which has been previously derived from the Alzheimer's Disease Neuroimaging Initiative (ADNI, adni.loni.usc.edu) on 283 subjects with mild cognitive impairment (MCI) who underwent serial MRIs at 1.5 T.^9^^,^^17^ The ADNI was launched in 2003 as a public–private partnership, led by Principal Investigator Michael W. Weiner, MD. For up-to-date information, see www.adni-info.org.

SPARE-AD is derived from patterns of regional brain atrophy (volume loss) captured by atlas warping methods and high-dimensional pattern classification using support vector machines (SVM) aiming to differentiate cognitively normal and Alzheimer’s disease subjects.^17–19^ We used cognitive decline as our outcome here, as measured by 2-year change from baseline values of the ADNI composite memory score ADNI-Mem.^20^ Average 2-year change from baseline for ADNI-Mem in the current study was −0.17 (standard deviation 0.49). The average age of the participants was 74.8 years (SD = 7.32), and 99 (35%) of participants were female. A more detailed description of demographics and clinical characteristics of patients has been published previously.^18^ We note that both the outcome and the disease status of the subjects studied here differ from the outcome and disease statuses that were used to build SPARE-AD. As a complementary analysis, we also examined conversion from MCI to Alzheimer’s disease as an outcome, employing time-to-event analysis methods, where we again used the SPARE-AD score as a radiomic predictor of interest. Approximately 60% of observations were censored.

As a second case study, we focussed on trials for GBM therapies in which the primary outcome is overall survival time after diagnosis. We analysed previously collected, anonymized data from 134 patients who were treated for newly diagnosed GBM at the Hospital of the University of Pennsylvania between 2006 and 2013. The median survival in this sample was 12 months, and survival data were assessed for all subjects with no loss to follow-up. The average age of patients in this study was 62.1 years (SD = 12.1), and 53 patients (40%) were female. Detailed demographics and a clinical description of these subjects have been previously published.^16^ All people with access to the data were on an institutional IRB. For this second case study, we investigated the use of cross-validated predictions of survival time based on radiomic analyses of pre- and post-contrast T_1_-weighted, T_2_-weighted and T_2_-fluid attenuated inversion recovery, diffusion, and perfusion MRI acquired pre-operatively at diagnosis. This GBM predictive model utilized an SVM model to differentiate short, medium and long survival.^16^

### Statistical analysis

All hypothesis testing was conducted assuming a 5% type I error rate and using two-sided alternatives. In lieu of data from a clinical trial, to explore the utility of our method, we employed plasmode simulation studies, in which all of our analyses were performed with datasets derived from ADNI and our GBM cohort, but we artificially generated treatment status and treatment effect. We also explored the method with synthetic data simulations, in which we artificially generated a theoretical radiomic predictor, a binary treatment indicator, and an outcome.

### Alzheimer’s disease study

For our Alzheimer’s disease plasmode simulation study, we used a continuous outcome and analysed our data with linear regression. To compare our method to a more classical analysis, we fit the following two models, where Equation (1) represents our method and Equation (2) represents a classical analysis:

(1) $$\;Y_{i}=\alpha +\beta X_{i}+\gamma A_{i}+\epsilon_{i}$$

(2) $$Y_{i}=\alpha +\gamma A_{i}+\epsilon_{i}$$

Here, $Y_{i}$ represents cognitive decline, defined as the difference between ADNI-Mem score observed at 2 years after baseline and ADNI-Mem score observed at baseline. $X_{i}$ represents the radiomic predictor, $A_{i}$ represents the treatment indicator, and $\epsilon_{i}$ represents random error. The parameters α and β are estimated from the data while γ is added in artificially, as described below. We note that these models are equivalent to ANOVA-CHANGE models as described in O’Connell et al.^21^

To conduct the simulation, we randomly split our data into two equal portions, one representing the source of a treated population and one representing the source of a control population. We then generated a sample treated arm and a sample control arm that we used for downstream analysis by sampling with replacement from the respective source samples. For the first group, indexed by $i=1,\;\ldots ,\frac{n}{2}$, we set our treatment indicator $A_{i}=0$ and record the observed outcome $Y_{i}$, as well as the value of the radiomic predictor $X_{i}$ at baseline. For the second group, indexed by $i=\frac{n}{2}+1,\;\ldots ,\;n$, we introduced a treatment effect γ, set our treatment indicator $A_{i}=1$, and again record outcome $Y_{i}$ and baseline radiomic predictor measurement $X_{i}$.

We repeated this process 1000 times, recording the *P*-value corresponding to the test for treatment effect each time. We calculated type I error rate and power as the percentage of times the treatment effect was significant at the α=0.05 level, where γ is set to 0 to assess type I error and a non-zero value to assess power. In order to quantify the sample size benefits from using this method, we repeated the above procedure for a range of sample sizes n and recorded the smallest n for which power reached 80%. We explored this for a range of hypothetical effect sizes, which was defined as γ divided by the standard deviation of the outcome $Y_{i}$.

We also performed a similar analysis with a time-to-event outcome, studying the time to conversion from MCI to Alzheimer’s disease measured in months. We analysed this outcome with the following accelerated failure time models, assuming a log-logistic distribution:

(1) $${\;Y}_{i}=\log(T_{i})=\alpha +\beta X_{i}+\gamma A_{i}+\sigma \epsilon_{i}$$

(2) $${\;Y}_{i}=\log{(T}_{i})=\;\alpha +\gamma A_{i}+\sigma \epsilon_{i}$$

Here, $X_{i}$ represents the radiomic predictor, and $A_{i}$ represents the binary treatment indicator. We introduce a multiplicative treatment effect on observed survival or censoring time in the treatment group and refer to this multiplier as the effect size. In order to mimic a 3-year clinical trial, we introduce end-of-study censoring at 36 months. We conducted the simulation study as described previously, assessing sample size benefits as the minimum number of participants for the study.

### Glioblastoma multiforme study

For our GBM plasmode simulation, we used survival outcomes and we assessed differences between treatment groups with and without adjustment for the radiomic prediction by assuming an accelerated failure time model. Specifically, we fit the following models:

(1) $${\;Y}_{i}=\log(T_{i})=\alpha +\beta X_{i}+\gamma A_{i}+\sigma \epsilon_{i}$$

(2) $${\;Y}_{i}=\log{(T}_{i})=\;\alpha +\gamma A_{i}+\sigma \epsilon_{i}$$

Here, $Y_{i}$ is $\log{(T}_{i})$, where $T_{i}$ represents the time to event, $X_{i}$ represents the radiomic predictor, $A_{i}$ represents the treatment indicator, and $\epsilon_{i}$ represents random error. In this study, we modelled $T_{i}$ using a log-logistic accelerated failure time model. We introduce a treatment effect and conduct the simulation study for this setting as described previously.

### Synthetic data simulation study

We start by discussing the continuous outcome case. We simulated data according to the following parametric form: $Y_{i}=2+4X_{i}+\;{\gamma A_{i}+\epsilon }_{i}$, where $X_{i}∼N\left(0,\;0.25\right)$ denotes a continuous predictor, $A_{i}$ is a binary treatment indicator simulated at random with$\;P\left(A_{i}=1\right)=0.5$, and $\epsilon_{i}∼N\left(0,\;1\right)$ is a random error term for i=1,…,n. We then introduced a treatment effect of γ for these subjects and assessed the significance of the treatment effect with a Wald test via linear regression. We repeated this process 1000 times, recording the *P*-value corresponding to the test for treatment effect each time. We calculated Type I error and power as the percentage of iterations in which the treatment effect was significant at the α=0.05 level, where we set γ to 0 to assess type I error and a non-zero value to assess power. In order to quantify the sample size benefits from using this method, we repeated the above procedure for a range of sample sizes n, corresponding to the total trial size, and recorded the smallest n for which power reaches 80%. We explored this for a range of hypothetical effect sizes, which we defined here as γ divided by the standard deviation of the outcome.

For the time-to-event outcome case, we followed a similar procedure. Here, the outcome $Y_{i}$ was simulated according to a Weibull distribution such that $Y_{i}=\log\left(T_{i}\right)=\;0.5+0.25X_{i}+4\epsilon_{i}$, where $T_{i}$ represents the time to event, $X_{i}∼N(0,\;1)$ denotes a continuous predictor, and $\epsilon_{i}$ is a random error term following an extreme value distribution with scale parameter of 4 for i=1,…,n, with total trial size n. Then, we introduced a treatment effect of γ with probability $P\left(A_{i}=1\right)=0.5$. We then used an accelerated failure time model to regress Y against A, testing for the treatment effect with a Wald test. We introduced end-of-study censoring at 36 months to mimic a 3-year clinical trial. We assessed power, Type I error, and sample size benefits as in the continuous outcome case.

### Data availability

The Alzheimer’s disease data used for this study are publicly available and were obtained from the ADNI database (http://adni.loni.ucla.edu/). Data collected for this study were approved under institutional review board protocol #825722 sponsored by the National Institutes of Health. The GBM data have been uploaded to TCIA, and should be available to the public shortly. For the purposes of the review process, the data are available as Supplementary File 1. Data for this study were collected under institutional review board-approved protocol #706564 sponsored by the National Institutes of Health.

## Results

**Figure 2:**
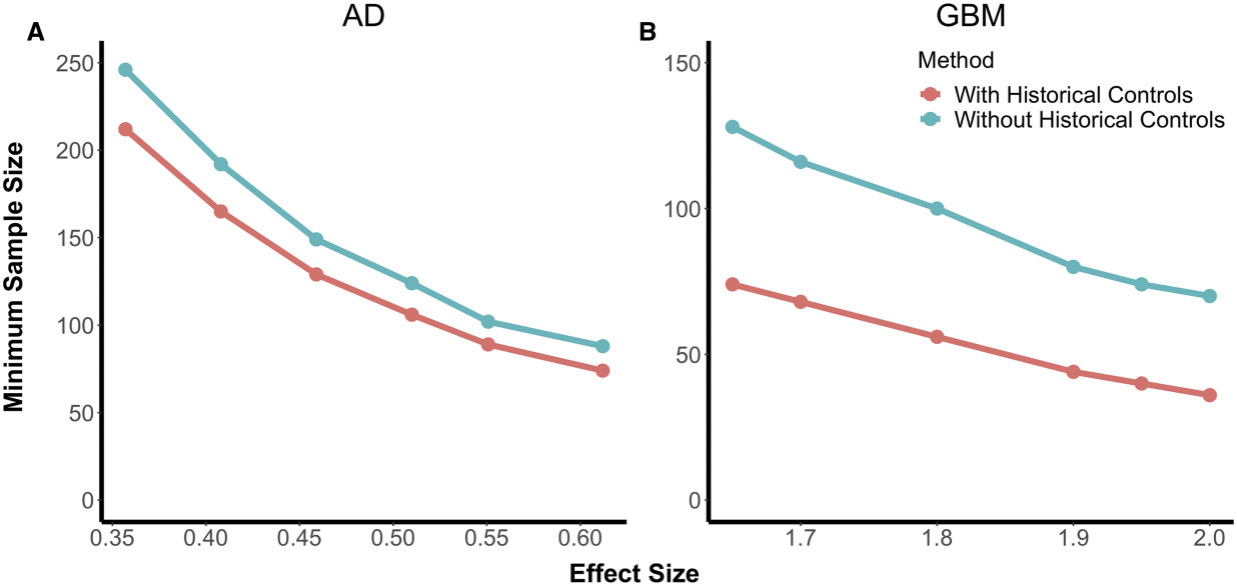
**Plasmode simulation results. Results from simulated studies under two scenarios.** With the addition of historical controls, the minimum required sample size for 80% power is markedly lower than using classical two-sample clinical trial analysis. These figures show minimum sample size (vertical axes) required to achieve 80% power for a range of effect sizes (horizontal axes) based on observed outcome and radiomic predictions. (**A**) shows the results from simulations for continuous outcome measures of cognition in our Alzheimer’s cohort from ADNI, analysed using a linear regression model with and without incorporation of the radiomic predictor (left). (**B**) shows the results from simulations for survival in our glioblastoma cohort, comprised of 134 patients who were treated for newly diagnosed GBM at the Hospital of the University of Pennsylvania between 2006 and 2013 and analysed with an accelerated failure time model with and without incorporation of the radiomic predictor. Note that the proposed method that leverages historical controls to build radiomic predictions (red) requires lower samples sizes than the classical approach (blue). Minimum required sample size was calculated as the smallest sample size that achieved 80% power as calculated by the percentage of Monte Carlo simulations with a non-zero treatment effect that were significant at the α = 0.05 level.

For both continuous and time-to-event outcomes, the proposed method consistently reduced the minimum required sample size n for a given level of power in clinical trial analyses (Fig. 2). In the Alzheimer’s disease plasmode simulations, where the outcome of interest is cognitive decline, with an effect size of 0.35, the total required sample size was 246 for the conventional analysis and 212 with the proposed historical control analysis. As the effect size increased, sample size requirements decreased for both approaches but decreased more rapidly for the conventional approach. In the GBM plasmode simulations, where the outcome of interest was survival time, at an effect size of 1.65, the total required sample size was 128 with the conventional analysis and 74 with the proposed historical control analysis.

Reductions in sample size requirements were greater for smaller effect sizes, so the benefit of historical controls declined as effect size increased. Type I error remained controlled throughout all experiments conducted. In the Alzheimer’s disease study with a continuous outcome, our proposed method resulted in a 14–16% decrease in the minimum required sample size. In the GBM study, our method reduced the required sample size by as much as 48%. Our simulations with fully synthetic data supported these findings.

Table 1 summarizes the effect of using our method on sample size across a range of power levels and effect sizes.

**Table 1:** Minimum required sample size for different powers and effect sizes

Cohort-outcome	Power	Effect size	Minimum sample size	
			With historical controls	Without historical controls
ADNI-continuous	0.8	0.40	174	200
		0.46	146	170
		0.61	74	88
	0.9	0.40	228	263
		0.46	172	204
		0.61	97	112
ADNI-survival	0.8	1.7	242	287
		1.8	206	254
		1.9	180	219
	0.9	1.7	—	—
		1.8	271	332
		1.9	238	292
GBM-survival	0.8	1.8	56	98
		1.9	44	82
		2	38	70
	0.9	1.8	74	130
		1.9	60	108
		2	50	90

In this table, we provide the minimum required sample size for both 80% and 90% power across a range of effect sizes in both our ADNI cohort and our cohort of patients with glioblastoma multiforme (GBM). For our ADNI dataset, because the radiomic predictor of interest is known to be an accurate predictor of MCI to Alzheimer’s disease conversion time, we also explored the utility of incorporating this method into a survival analysis.

**Figure 3:**
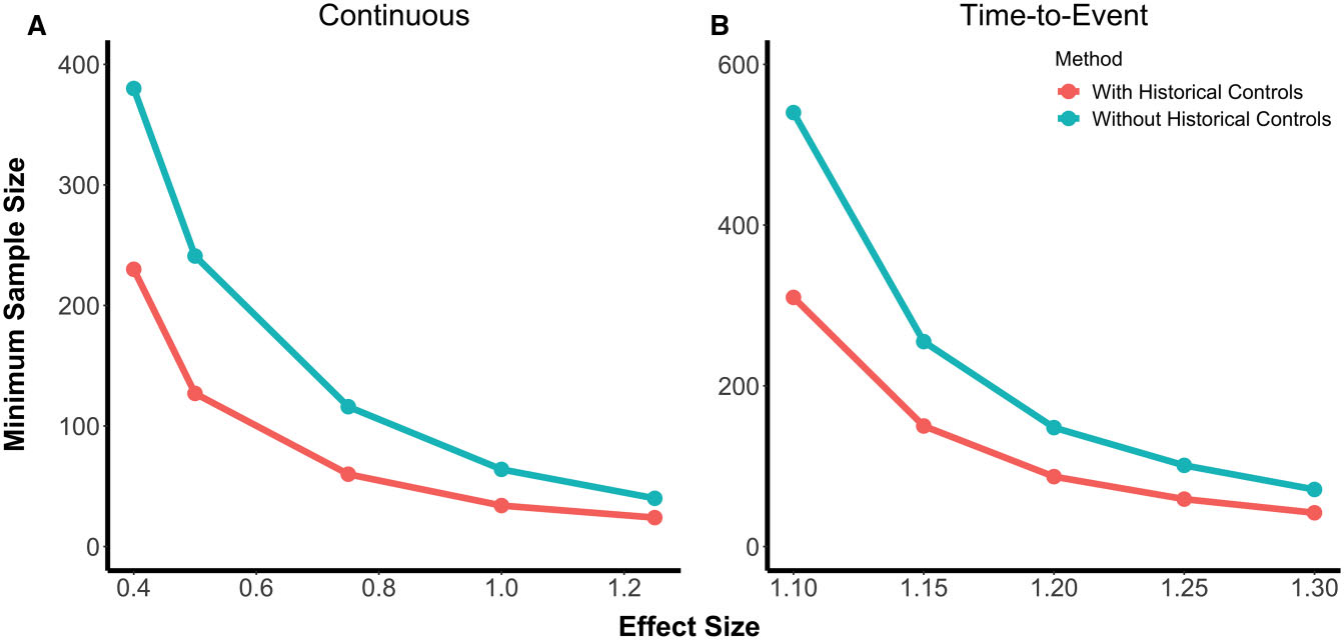
**Synthetic data simulation results.** Results from simulated studies with synthetic data generated to be homogenous across all cohorts except for random error and treatment status. These figures show the minimum sample size required to achieve 80% power for a range of effect sizes, where minimum sample size was calculated as the smallest sample size for which at least 80% of Monte Carlo simulations with a non-zero treatment effect were significant at the α = 0.05 level. (**A**) shows the results for simulations with a continuous outcome, analysed using linear regression with and without incorporation of the radiomic predictor, and (**B**) shows the results for simulations with a survival outcome, analysed using an accelerated failure time model with and without incorporation of the radiomic predictor. In both cases, the proposed method that leverages historical controls in the form of radiomic predictions (red) requires lower sample sizes than the classical approach (blue).

We also explored our method with synthetic data simulations (Fig. 3). Results for these simulations were similar to those from the plasmode simulation studies. With the synthetic data simulations, we noticed even greater sample size gains with the use of our proposed methodology. In settings with continuous outcomes, at the smallest effect size that we studied of 0.4, the total required sample size was 380 when using the conventional analysis and 230 when properly incorporating information from historical controls. As in the plasmode studies, this reduction became less pronounced as the effect size increased. This is partly due to a more rapid decrease in required sample size under the conventional approach than for the proposed method. In the time-to-event outcome simulations, at the smallest effect size studied of 1.1, the total required sample size was 540 with the conventional analysis and 310 with the proposed historical control analysis.

## Discussion

We have shown that individualized machine-learning-based imaging biomarkers can be useful tools in clinical trial analysis when the necessary information is available, offering decreased sample size requirements for a given effect size. The novelty of this method arises from the incorporation of individualized predictions based on powerful predictive algorithms which lend power to the detection of an average treatment effect due to targeting of the individuals in a given clinical trial. As robust neuroimaging biomarkers derived via machine learning models become more available, the historical datasets that can be analysed with those models grow in size. These changes are expected to strengthen the radiomic prediction models like the ones used in this study.

Our incorporation of the radiomic predictor relies on the existence of previously developed radiomic predictors, which for the purposes of this paper, we theorize as having been trained on a historical cohort. Because the radiomic predictor has been previously trained on a historical cohort, the models that we fit to analyse a current trial inherently incorporate information from historical controls. When we do not include the radiomic predictor into the clinical trial analysis, we do not incorporate information from historical controls. We generate values of the radiomic predictor for the current sample using the model that was developed with a previous cohort. For our simulations, we assume that the current trial is being run on an experimental drug and the goal is to show superiority.^21^ Patients enrolled in a clinical trial or a cohort study may not be representative of patients in a population of interest. Differences between these populations are the result of the explicit inclusion/exclusion criteria of a clinical trial as well as the indirect differences between patients who are willing to volunteer for a clinical trial and those who are not.^22^ Event rates can also be higher in a cohort study than in a clinical trial, potentially due to these same biases. However, randomization of the current trial participants ensures that even if the historical control population is different from the trial population in important ways we would not realize inflated type I error. The differences may however impact the predictive performance of the radiomic predictor for the outcome of interest, which could thus impact statistical power of the proposed methodology and attenuate the sample size benefits.

In the event that a primary analysis of an endpoint does not yield statistically significant results, this technique could potentially be used in a sensitivity analysis to aid interpretation. Performance of a secondary analysis looking to draw conclusions about the efficacy of a treatment could result in increases of type 1 error and thus spurious decisions, but incorporation of this technique into an exploratory analysis aimed at characterizing the impact of baseline heterogeneity on inference could illuminate important phenomena that would otherwise be missed.

We note that unexplained biological heterogeneity among the cohorts under study may have attenuated the power gains that were observed. To assess the potential gains of using this method in cases where the degree of biological heterogeneity explained was modifiable, we conducted simulation studies with data generated so as to be homogenous except for random error and treatment status (Fig. 3). In that setting, power gains and subsequent sample size reductions were much more dramatic.

Here, we used two previously developed biomarkers, one of which was trained to classify an outcome different from the target of the clinical trial analysis, and the other of which was trained to classify the same outcome as the clinical trial analysis. While both predictors offered gains in sample size reduction, the predictor built specifically for the outcome of interest in the clinical trial performed better and offered more substantial gains. We expect that the gains in power will likely be larger when the model is trained to predict the primary outcome of the clinical trial, though this needs to be empirically tested across a range of applications.

This approach is not limited to radiomic predictors. It can be applied to a broad array of factors associated with the outcomes of interest in a clinical trial, such as clinical variables, blood or cerebrospinal fluid-based biomarkers, or genomic markers. The choice between use of biomarker-only prediction models as opposed to clinical and biomarker prediction models can be decided on a case-by-case basis, depending on the hypothesis of interest in a given study. In general, the more robust the associations among the predictors and the outcome of interest, the greater the anticipated gains in power or reduction in sample size required for a specified level of power.

The approach proposed in this paper has some limitations. First, the use of radiomic predictions can be hindered by the cost of collecting imaging data.^23^ Though many modern clinical trials of neurologic disease now incorporate baseline MR imaging into their protocol,^24–30^ especially in those of Alzheimer’s disease and GBM, which we use as examples in this manuscript, imaging remains expensive and potentially extraneous for the study of certain diseases. This method is only helpful when baseline imaging is available, and in the absence of baseline imaging for participants of a new study, incorporation of radiomic predictors through prior scans from unknown length of time prior to the new study may result in attenuated benefits of statistical power and imperfect characterization of disease load. This can also impact the accuracy of the sample size calculation in that incorporation of data that does not reflect true baseline heterogeneity at the beginning of a new study can increase uncertainty in the analysis.

Furthermore, reductions in sample size requirement depend upon the strength of the prediction model. In this study, both radiomic predictors considered were built based on SVMs, but other machine learning techniques such as deep convolutional neural networks may provide more predictive power.^31^ In addition, gains in power for the primary outcome will be associated with gains in power for secondary outcomes only to the extent that predictions from the prediction model are associated with the secondary outcomes. This will likely be determined by the degree of correlation between the primary outcomes and a set of secondary outcomes. In principle, within a single trial, separate prediction models could be developed for two or more co-primary endpoints. Incorporation of this method into randomized trials with more complex designs, such as one incorporating stratification by confounders or a one-arm trial, requires further statistical research.

Finally, if a radiomic predictor is trained on data sampled from a different population than that which is studied in the current trial, the improvements in statistical power may be less pronounced. However, due to the randomization in the study, the type I error rate is expected to be maintained and internal validation or calibration of the predictive model is possible using data from the control arm of a clinical trial.

The key conclusion arising from our study is that machine-learning-based predictive models can be used to effectively improve the statistical power of clinical trials by leveraging the wealth of information available in neuroimaging data to generate personalized predictions of outcome. Radiomic predictors are seldom incorporated into clinical trial analyses, but we have demonstrated that when the necessary information is available, they can be a powerful tool, especially when evaluating therapies for rare diseases such as GBM or heterogenous diseases with long and slow progressions that require many years of patient follow-up such as Alzheimer’s disease.

## Supplementary material

Supplementary material is available at *Brain Communications* online.

## Supplementary Material

fcab264_Supplementary_Data

## Abbreviations

ADNI =: Alzheimer's Disease Neuroimaging Initiative;
GBM =: glioblastoma multiforme;
MCI =: mild cognitive impairment;
SVM =: support vector machines
