# Supplementary material for: Leveraging machine learning predictive biomarkers to augment the statistical power of clinical trials with baseline magnetic resonance imaging
Source: Brain Commun. 2021 Nov 3;3(4):fcab264. doi: 10.1093/braincomms/fcab264 (PMC8600962; doi:10.1093/braincomms/fcab264)
Supplement: fcab264_Supplementary_Data [file fcab264_supplementary_data.pdf]

S1File

	Survival	SPI	Age	Sex
1	26.33333333	0.999935228	58	F
2	18.56666667	0.999950175	73	F
3	1.466666667	0.360199942	66	M
4	11.76666667	0.392891057	67	M
5	15.26666667	0.632037232	42	F
6	3.133333333	0.000179369	65	M
7	0.766666667	0.154925606	72	F
8	15.23333333	0.632577701	63	M
9	1.866666667	0.507015306	80	F
10	5.133333333	0.070219754	67	F
11	9.2	0.024855696	86	F
12	19.26666667	0.999939451	54	M
13	2	0.333333333	85	M
14	8.333333333	0.629357898	59	F
15	8.266666667	0.642818743	46	M
16	0.7	8.47018E-05	86	F
17	6.066666667	0.653061224	52	M
18	11.53333333	0.336750717	79	M
19	12.46666667	0.5	54	M
20	9.233333333	0.50009229	83	F
21	19.53333333	0.862618003	64	F
22	10.03333333	0.300387875	61	F
23	0.1	0.000100636	53	M
24	13.06666667	0.5000193	50	M
25	16.16666667	0.499985446	85	M
26	14.13333333	0.429281261	49	F
27	16.83333333	0.663250708	74	M
28	1.466666667	0.077425464	77	F
29	19.43333333	0.653037326	76	F

30	3.666666667	0	72	F
31	7.666666667	0.64278628	69	F
32	14.6	0.642857143	49	F
33	6.066666667	0.653026762	56	M
34	4.6	6.4772E-05	53	F
35	12.93333333	0.532870882	52	F
36	3.2	8.47018E-05	80	M
37	32.93333333	0.769766521	43	M
38	4.866666667	0.155397615	60	F
39	0.5	0.142938523	75	M
40	6.666666667	0.469387755	88	F
41	22.4	0.832737162	55	F
42	4.533333333	6.4772E-05	56	M
43	1.7	0.163265306	57	F
44	5.6	8.13802E-05	79	M
45	2.666666667	0.176128389	59	F
46	9.066666667	0.663265306	63	M
47	33.6	0.86013517	52	M
48	4.066666667	0.352272227	82	M
49	2.433333333	0.152397339	76	M
50	0.533333333	0.415789915	83	M
51	12.63333333	0.333333333	47	M
52	2.6	9.05615E-05	72	F
53	32	0.504853334	52	F
54	9.7	0.499979451	61	F
55	20.76666667	0.83652543	33	M
56	38.1	0.857142857	54	M
57	20.86666667	0.496598639	53	M
58	11.6	0.333368211	57	M
59	18.7	0.857078085	69	M
60	16.7	0.333361971	77	M
61	16.6	0.5	59	M

62	29.4	0.633753196	54	M
63	9.033333333	0.34375	68	M
64	18.46666667	0.734448568	54	M
65	16.96666667	0.5	57	F
66	39	0.793749173	40	M
67	45.26666667	0.946156514	43	F
68	14.16666667	0.352526359	50	M
69	17	0.357142857	49	F
70	15.13333333	0.663265306	54	M
71	23.66666667	0.999951267	64	F
72	13.56666667	0.32423813	65.12	F
73	50.13333333	0.821813015	22.04	M
74	16.73333333	0.5	66.86	M
75	23.56666667	0.732490869	60.56	M
76	12.13333333	0.360284644	56.26	M
77	22.13333333	0.492853003	58.44	M
78	68.26666667	0.84375	64.55	F
79	12	0.34375	52.50	M
80	11.96666667	0.360234771	65.69	M
81	19.93333333	0.832562503	62.09	M
82	14.93333333	0.507015306	63.52	M
83	12.63333333	0.653061224	63.45	M
84	17.93333333	0.663265306	50.83	F
85	6.5	0.499997576	58.78	M
86	14.56666667	0.330054666	63.49	M
87	7.466666667	0.576293367	57.69	M
88	22.16666667	0.687408953	54.07	M
89	26.03333333	0.999856469	60.25	F
90	18.76666667	0.632653061	53.38	M
91	13.2	0.403739363	64.93	F
92	13.73333333	0.489795918	46.62	F
93	2.466666667	7.03188E-05	44.72	M

94	22.43333333	0.999959241	65.99	M
95	12.3	0.659440818	56.56	M
96	14.83333333	0.418144104	59.10	M
97	14.23333333	0.505227971	61.44	M
98	28.2	0.442312807	68	M
99	7.766666667	0.511915679	75	M
100	4.066666667	0.495879996	59	M
101	4.966666667	0.593109564	67	M
102	20.73333333	0.5	45	M
103	5.166666667	0.520408163	65	M
104	5.566666667	0.735901821	59	F
105	16	0.479677868	64	F
106	38.4	0.349411031	80	F
107	29.3	0.445796777	73	F
108	3.7	0.079309106	75	F
109	16.43333333	0.497640165	62	F
110	8.8	0.685903548	64	M
111	9.4	0.237195244	72	F
112	2.233333333	0.401772427	70	M
113	11.4	0.529435915	52	M
114	1.233333333	0.465364809	75	F
115	81.7	0.555422599	37	F
116	15.2	0.433973455	59	F
117	9.7	0.565478838	68	M
118	35.7	0.609864822	56	M
119	9.266666667	0.437567528	54	M
120	1.8	0.304131329	80	M
121	5.266666667	0.300876474	70	M
122	1.9	0.355184778	78	F
123	19.43333333	0.774131193	57	F
124	86.83333333	0.842490553	61	M
125	0.8	0.415260917	62	M

126	10.9	0.445578781	68	F
127	7.6	0.506933748	44	M
128	8.2	0.320865351	68	M
129	21.6	0.412047266	47	F
130	81.7	0.841465242	48	M
131	4.866666667	0.344619537	70	M
132	1.5	0.17295498	71	M
133	5.566666667	0.228400409	76.14	F
134	3.533333333	0.475414687	77.72	M
